# Identification of genes and transcription factors associated with glucocorticoid response in lens epithelial cells

**DOI:** 10.3892/mmr.2015.3308

**Published:** 2015-02-06

**Authors:** DING ZHOU, YI ZHANG, LISHAN WANG, YUNDUAN SUN, PING LIU

**Affiliations:** 1 Department of Ophthalmology, The First Affiliated Hospital of Harbin Medical University, Harbin, Heilongjiang 150001, P.R. China; 2 Bio-X Center, Shanghai Jiao Tong University, Shanghai 200230, P.R. China

**Keywords:** lens epithelial cells, glucocorticoid, dexamethasone, function enrichment analysis, transcription factor

## Abstract

Prolonged glucocorticoids (GCs) treatment may lead to the formation of posterior subcapsular cataracts. The present study aimed to investigate differential gene expression in lens epithelial cells (LECs) in response to GCs using DNA microarray profiling. The gene expression profile of GSE13040 was downloaded from the Gene Expression Omnibus database, which includes 12 human LECs treated with vehicle or dexamethasone (Dex) for 4 or 16 h with six samples at each time period, of which three samples were treated with vehicle (control group) and three samples were treated with Dex (Dex group) at each time point. The differentially expressed genes (DEGs) were identified between the control group and the Dex group at each time period with the thresholds of P<0.05 and |logFC|>1. The DEGs were further analyzed using bioinformatics methods. Firstly, DEGs were subject to a hierarchical cluster analysis. Subsequently, the functional enrichment analysis was performed for the common DEGs between the two time periods. Finally, the transcription factors and binding sites of DEGs associated with response to GC stimulus were analyzed. A total of 696 and 949 DEGs were identified at 4 h and 16 h, respectively. Hierarchical cluster analysis revealed that DEG expression was higher in the Dex group than in the control group (P<0.05). A total of 13 significant functions were enriched for the 72 common DEGs at the two time periods. Chemokine (C-C motif) ligand 2 (CCL2), dual-specificity phosphatase-1 (DUSP1) and FAS were associated with the response to GC stimulus and the transcription factor c-Jun bound to promoter regulation regions of CCL2, DUSP1 and FAS. In conclusion, the transcription factors and binding sites of DEGs associated with the response of LECs to GCs may provide potential gene targets for designing and developing drugs to protect against GC-induced cataract formation.

## Introduction

Glucocorticoid (GCs) steroid hormones are used in the treatment of diseases, including rheumatoid arthritis, asthma and various ocular diseases. It has been widely reported that prolonged treatment with GCs can lead to the formation of posterior subcapsular cataracts (1–3). Although numerous attempts have been made to increase understanding of this, the mechanism underlying GC-induced cataract formation remains to be elucidated (4,5).

GCs have important roles in numerous biological processes, including regulation of anti-inflammatory activity and immunosuppressive action (6,7). GCs exert their effects through binding to GC receptors (GR), which modulate the expression of target genes (8,9). Alternatively, GCs have been proposed to act on the lens indirectly through mechanisms involving oxidative stress and depletion of glutathione (10,11). Global gene profiling was performed to analyze novel GC-induced changes in the gene expression of human lens epithelial cells (LECs) (12). Following this study, pathway analysis was performed in immortalized and primary human LECs and the results demonstrated that GC treatment of LECs activated the GR to modulate the expression of mitogen-activated protein kinase and phosphatidylinositol-3-kinase/AKT regulators (13).

To improve the understanding of the mechanism involved in the formation of cataracts, GC’s induction of vascular barrier function requires elucidation. GCs combine with a cytoplasmic receptor that alters gene expression in two ways. One way is dependent on the receptor binding directly to DNA and acts as a transcription factor (positively or negatively). The other is dependent on its binding to and interfering with other transcription factors (14). Transcription factor p54 is essential for GC-mediated expression of occludin, claudin-5 and vascular barrier induction, and the p54/PSF heterodimer may contribute to normal blood-retinal barrier induction *in vivo* (15). Thus, it is necessary to elucidate the transcription factors that are activated in response to GCs.

The present study aimed to identify differentially expressed genes (DEGs) and their common transcription factors in order to gain a novel insight into the mechanism of action of GCs in LECs.

## Materials and methods

### Affymetrix microarray data

The transcription profile of GSE3040 was obtained from the gene expression omnibus (GEO, http://www.ncbi.nlm.nih.gov/geo/) database, which is based on the GPL96 [HG-U133A] Affymetrix Human Genome U133A Array (Affymetrix Inc., Santa Clara, CA, USA). There were 12 samples of human LECs treated with vehicle or dexamethasone (Dex) at 4 and 16 h. At each time period, there were six samples, of which three samples were treated with vehicle (control group) and three samples were treated with Dex (Dex group). Freshly isolated human LECs were obtained from capsulorhexis specimens following surgery, these were the original cells used in the GEO (12).

### Data preprocessing and DEG analysis

The GSE3040 datasets were converted into expression values and pre-processing, including background correction and quartile data normalization were performed using the robust multiarray average algorithm (16) with default parameters in the R language affy package (http://www.bioconductor.org/) (17,18). The linear models for microarray analysis (Limma) package in the R language (www.bioconductor.org/packages/release/bioc/html/limma.html) (19) were used to identify DEGs by performing Student’s t-test on the samples. A fold change value >1 and P<0.05 were selected as the cut-off criteria.

### Hierarchical cluster analysis of DEGs

Gene hierarchical cluster analysis of DEGs was performed using the Pearson correlation coefficient algorithm (20) in cluster 3.0 (21).

### Functional enrichment analysis of common DEGs

The Database for Annotation, Visualization and Integrated Discovery (DAVID; http://david.abcc.Ncifcrf.gov/) (22), a high-throughput and integrated data-mining environment, analyzes gene lists derived from high-throughput genomic experiments. After the common DEGs were selected, DAVID was used to identify over-represented gene ontology (GO; http://www.geneontology.org/) categories in biological processes based on the hypergeometric distribution. The GO terms with a value of P<0.05 were selected as significantly enriched DEGs.

### Transcription factors and binding site analysis

A transcription factor is a protein, which binds to specific DNA sequences. The TRANSFAC database comprising information about transcription factors, target genes and binding sites has been developed (23). The TRANSFAC database was used to screen transcription factors and binding sites on DEGs in response to GCs.

## Results

### DEG analysis

The publicly available microarray dataset, GSE3040, was obtained from the GEO database. Student’s t-test was used to identify genes specifically differentially expressed at 4 and 16 h with the cut-off criteria of P<0.05 and fold change >1. The results revealed that 696 and 949 genes at 4 and 16 h, respectively, exhibited significant differential expression.

### Hierarchical cluster analysis of DEGs between Veh and Dex samples at two time periods

**Figure 1:**
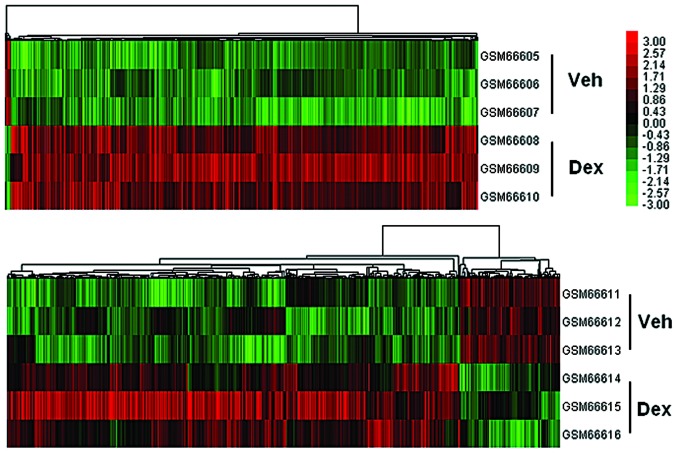
Heat map of cluster analysis of differentially expressed genes between Veh and Dex samples. Green, downregulated genes; and Red, upregulated genes. Dex, dexamethasone; veh, vehicle.

As indicated using hierarchical cluster analysis, the expression levels of DEGs were markedly increased in Veh samples compared with that of the Dex group, at 4 and 16 h (Fig. 1).

### Set comparison of DEGs between two time periods

**Figure 2:**
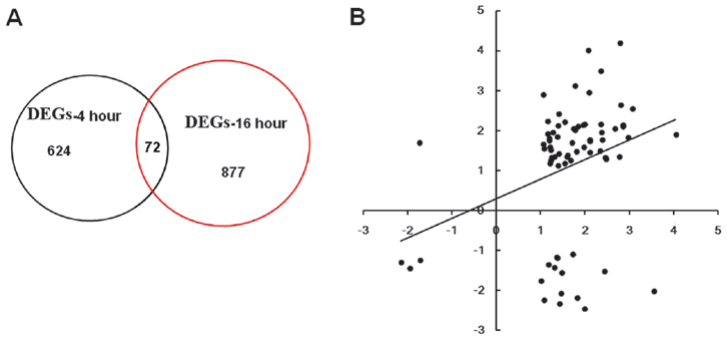
DEGs in lens epithelial cells in response to dexamethasone. (A) Venn diagram depicting common DEGs. The black and red circles represent DEGs at 4 h and 16 h treated with dexamethasone, respectively. (B) Correlation of gene expression of common DEGs between 4 h and 16 h. X-axis, log(FC) of DEGs at 4 h; and Y-axis, log (FC) of DEGs at 16 h. DEGs, differentially expressed genes; FC, fold change.

DEGs set at 4 and 16 h were compared and presented as a Venn diagram (Fig. 2A). There were 72 common DEGs. The expression folds of 72 DEGs at 4 and 16 h are shown in Fig. 2B. The results revealed that the gene expression trend at 4 h was the same as that at 16 h.

### GO enrichment analysis

**Figure 3:**
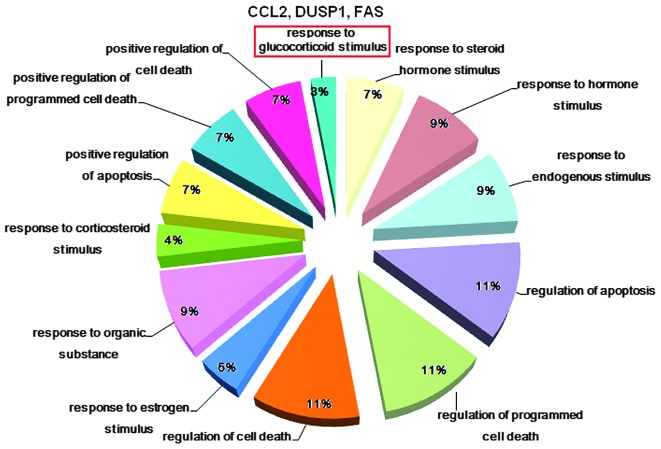
Pie chart depicting the hierarchical clustering of enriched functions involving common differentially expressed genes in the lens epithelial cells treated with dexamethasone.

To gain further insight into the function of genes in our interaction network, the online biological classification tool DAVID was used. A total of 13 significant GO function enrichment nodes were obtained and the distributions of genes is shown in Fig. 3. As Table I demonstrates, Chemokine (C-C motif) ligand 2 (CCL2), dual-specificity phosphatase-1 (DUSP1) and FAS were associated with the function of response to GC stimulus (P=0.0496906). Expression of CCL2 was downregulated, and DUSP1 and FAS were upregulated at 4 and 16 h (P<0.05).

### Transcription factor analysis

**Figure 4:**
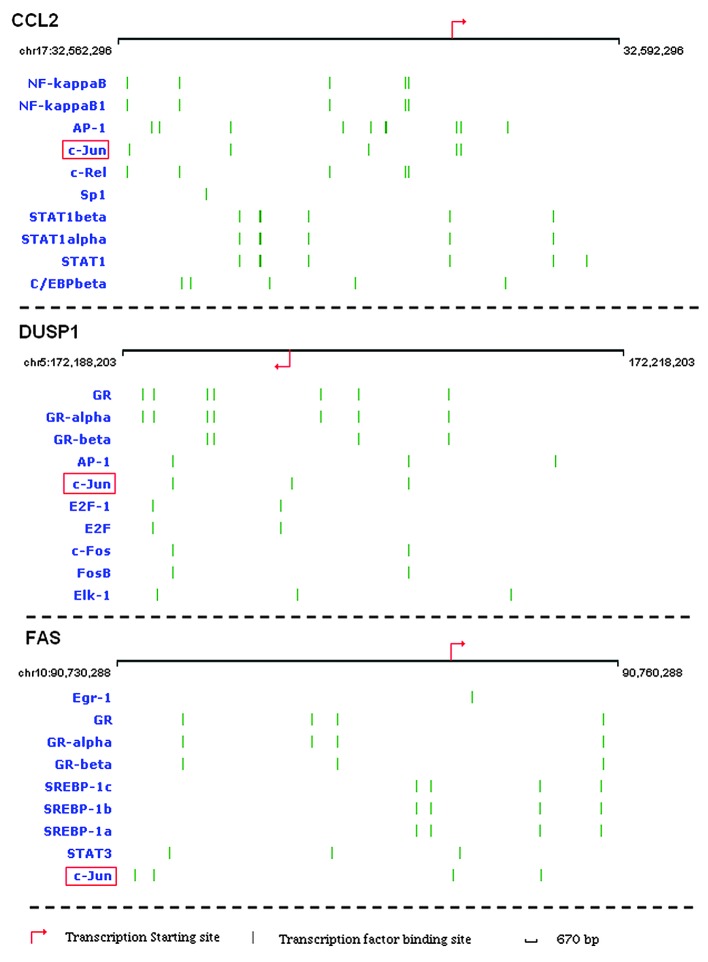
Map of transcription factor binding on the promoter regulatory regions of CCL2, DUSP1 and FAS. Green bars represent the binding sites. Red arrows represent transcription initiation sites and directions. CCL2, chemokine (C-C motif) ligand 2; DUSP1, dual specificity protein phosphatase 1.

Using the TRANSFAS database, the transcription factors and binding sites, which were associated with the three GC response genes, CCL2, DUSP1 and FAS, were assessed. As Fig. 4 demonstrates, c-Jun binds to the promoter regulatory regions of these three genes and was the common transcription factor (Fig. 4).

## Discussion

GCs have been used in clinical treatment for decades; however, prolonged GC treatment may lead to the formation of cataracts (24). In the current study, using DNA microarray analysis, the gene expression profiles of human LECs treated with Dex or vehicle were analyzed. A total of 13 significant GO functions were identified and CCL2, DUSP1 and FAS genes were associated with a response to GC stimulus. The transcription factor that binds to CCL2, DUSP1 and FAS were also analyzed. The results demonstrated that c-Jun was a common transcription factor between these genes.

CCL2 is also known as monocyte chemotactic protein-1 and is secreted by endothelial cells, fibroblasts and monocytes (25). It has been reported that CCL2 expression and macrophage accumulation were inhibited by treatment with Dex in cholesterol-fed rabbits (26). GRs may bind specifically to CCL2 mRNA and the inflammatory response of the GR was mediated by regulation of CCL2 mRNA stability (27). CCL2 was detected in the sample obtained from patients following cataract surgery (28). DUSP1 is a member of the threonine-tyrosine dual-specificity phosphatases (29). Increased expression of GILZ mRNA and DUSP1 mRNA and protein was observed in immortalized and donor immortalized primary LECs (13). The induction of DUSP1 is dependent on the GR and typically occurs within ≤1 h (30). The FAS receptor is an important cell surface receptor protein of the tumor necrosis factor receptor family (31). Yang *et al* (32) reported that FAS ligand expression was inhibited by retinoic acid and GCs.

In the present study, c-Jun was observed to bind the promoter regulatory regions of CCL2, DUSP1 and FAS. The c-Jun gene encodes a basic region-leucine zipper transcription factor implicated in numerous cellular processes. C-Jun regulates gene expression and cell function by being involved in the formation of a variety of dimeric complexes, which exhibit high affinity sequence specific DNA-binding activity (33). It has been reported that c-Jun attenuated MG132-induced activation of activator protein-1 and expression of CCL2 (34). The Hepatitis C virus core protein expression activated MAP kinase phosphatase, increased DUSP1 expression and increased cell proliferation, which was accompanied by an activation of c-Jun (35). The expression of dominant-negative c-Jun in melanoma cells efficiently increased Fas expression (36). The present results demonstrated that c-Jun may be the critical transcription factor, which affected gene expression in LECs in response to GCs.

In conclusion, the gene expression profiles of LECs following GC treatment were analyzed using bioinformatics analysis and it was found that CCL2, DUSP1 and FAS are involved in the response to GC stimulus. The transcription factor c-Jun, when bound to CCL2, DUSP1 and FAS, may affect their expression. CCL2, DUSP1, FAS and transcription factor c-Jun may be used as specific therapeutic molecular targets in order to treat cataracts induced by GCs. However, further studies are required to confirm the present results.

## Figures and Tables

**Table I:** Enriched Gene Ontology terms of the common differentially expressed genes at 4 h and 16 h (P<0.05).

Term	Function	Count	P-value	Genes
GO:0048545	Response to steroid hormone stimulus	7	0.000240124	KCNMA1, CCL2, DUSP1, LEPR, ESR1, FAS, CD24
GO:0009725	Response to hormone stimulus	9	0.000255726	KCNMA1, CCL2, DUSP1, LEPR, ESR1, FOXC2, FAS, CD24, STAT1
GO:0009719	Response to endogenous stimulus	9	0.000494549	KCNMA1, CCL2, DUSP1, LEPR, ESR1, FOXC2, FAS, CD24, STAT1
GO:0042981	Regulation of apoptosis	12	0.000955092	KCNMA1, PRUNE2, CCL2, DUSP1, MCL1, SOS2, ESR1, FOXC2, FAS, CD24, STAT1, ANGPTL4
GO:0043067	Regulation of programmed cell death	12	0.001035716	KCNMA1, PRUNE2, CCL2, DUSP1, MCL1, SOS2, ESR1, FOXC2, FAS, CD24, STAT1, ANGPTL4
GO:0010941	Regulation of cell death	12	0.001067374	KCNMA1, PRUNE2, CCL2, DUSP1, MCL1, SOS2, ESR1, FOXC2, FAS, CD24, STAT1, ANGPTL4
GO:0043627	Response to estrogen stimulus	5	0.001353113	KCNMA1, DUSP1, LEPR, ESR1, CD24
GO:0010033	Response to organic substance	10	0.005300596	KCNMA1, CCL2, DUSP1, MCL1, LEPR, ESR1, FOXC2, FAS, CD24, STAT1
GO:0031960	Response to corticosteroid stimulus	4	0.006933831	KCNMA1, CCL2, DUSP1, FAS
GO:0043065	Positive regulation of apoptosis	7	0.013655682	KCNMA1, PRUNE2, DUSP1, SOS2, FAS, CD24, STAT1
GO:0043068	Positive regulation of programmed cell death	7	0.01409138	KCNMA1, PRUNE2, DUSP1, SOS2, FAS, CD24, STAT1
GO:0010942	Positive regulation of cell death	7	0.01438722	KCNMA1, PRUNE2, DUSP1, SOS2, FAS, CD24, STAT1
GO:0051384	Response to glucocorticoid stimulus	3	0.049690594	CCL2, DUSP1, FAS

CCL2, chemokine (C-C motif) ligand 2; DUSP1, dual specificity protein phosphatase 1.
